# Krüppel-like factor 12 regulates aging ovarian granulosa cell apoptosis by repressing SPHK1 transcription and sphingosine-1-phosphate (S1P) production

**DOI:** 10.1016/j.jbc.2023.105126

**Published:** 2023-08-04

**Authors:** Chun-Xue Zhang, Yu-Ling Lin, Fei-Fei Lu, Li-Na Yu, Yang Liu, Ji-Dong Zhou, Na Kong, Dong Li, Gui-Jun Yan, Hai-Xiang Sun, Guang-Yi Cao

**Affiliations:** 1 Center for Reproductive Medicine and Obstetrics and Gynecology, Nanjing Drum Tower Hospital Clinical College of Nanjing Medical University, Nanjing, China; 2 State Key Laboratory of Reproductive Medicine and Offspring Health, Nanjing Medical University, Nanjing, China; 3 State Key Laboratory of Pharmaceutical Biotechnology, Nanjing University, Nanjing, China

**Keywords:** KLF12, SPHK1, ovary, granulosa cell, apoptosis

## Abstract

Oxidative stress triggered by aging, radiation, or inflammation impairs ovarian function by inducing granulosa cell (GC) apoptosis. However, the mechanism inducing GC apoptosis has not been characterized. Here, we found that ovarian GCs from aging patients showed increased oxidative stress, enhanced reactive oxygen species activity, and significantly decreased expression of the known antiapoptotic factor sphingosine-1-phosphate/sphingosine kinase 1 (SPHK1) in GCs. Interestingly, the expression of Krüppel-like factor 12 (KLF12) was significantly increased in the ovarian GCs of aging patients. Furthermore, we determined that KLF12 was significantly upregulated in hydrogen peroxide–treated GCs and a 3-nitropropionic acid–induced *in vivo* model of ovarian oxidative stress. This phenotype was further confirmed to result from inhibition of SPHK1 by KLF12. Interestingly, when endogenous KLF12 was knocked down, it rescued oxidative stress–induced apoptosis. Meanwhile, supplementation with SPHK1 partially reversed oxidative stress–induced apoptosis. However, this function was lost in SPHK1 with deletion of the binding region to the KLF12 promoter. SPHK1 reversed apoptosis caused by hydrogen peroxide–KLF12 overexpression, a result further confirmed in an *in vitro* ovarian culture model and an *in vivo* 3-nitropropionic acid–induced ovarian oxidative stress model. Overall, our study reveals that KLF12 is involved in regulating apoptosis induced by oxidative stress in aging ovarian GCs and that sphingosine-1-phosphate/SPHK1 can rescue GC apoptosis by interacting with KLF12 in negative feedback.

With increasing age, female fertility gradually decreases, mainly manifested as increased apoptosis of ovarian granulosa cells (GCs), accelerated follicular atresia, and significantly decreased quantity and quality of oocytes, eventually leading to ovarian function decline (1, 2, 3). Oxidative stress triggered by aging can impair ovarian function by inducing ovarian GC apoptosis. The follicle, composed of oocytes and surrounding GCs, is the main endocrine and reproductive unit in the female ovary. As an important part of follicles, GCs provide nutritional and mechanical support to oocytes, which is crucial for follicular development and maintenance of ovarian microenvironment homeostasis (4). Abnormal behavior of GCs, such as apoptosis, can lead to increased follicular atresia and a decline in ovarian function (5, 6).

The expression of related proapoptotic genes increased in ovarian GCs of aged mammals, whereas the expression of some oxidoreductase activity–related genes was significantly downregulated, indicating that the antioxidative stress ability of GCs decreased and the apoptosis level increased (7). Estrogen secreted by GCs not only promotes follicular growth and development but also promotes GC survival through paracrine and autocrine signaling and protects against hydrogen peroxide (H_2_O_2_)–induced GC apoptosis (8, 9). Oxidative stress caused by the gradual accumulation of reactive oxygen species (ROS) in the ovary is one of the important reasons for ovarian aging. Mechanistically, GC proliferation and apoptosis during follicular development are regulated by PI3K–Akt–FOXO1 signaling (10, 11).

Sphingosine-1-phosphate (S1P), a bioactive metabolic product of sphingolipids, has been reported to protect GCs from apoptosis induced by oxidative stress *via* the PI3K–Akt–FOXO1 signaling pathway (12, 13, 14). S1P is produced by SPHK1, an important rate-limiting enzyme for maintaining sphingolipid balance, in the cytoplasm and plays a role in cell apoptosis (15). Long-acting oral S1P mimics (FTY720, fingolimod) have recently been developed and applied to female patients with multiple sclerosis (16, 17). However, the regulation of SPHK1–S1P signaling and upstream regulatory molecules on GC apoptosis is rarely reported. Hence, exploring the mechanism of oxidative stress–induced GC apoptosis provides a possible new target for saving abnormal apoptotic GCs, which has important clinical significance for improving ovarian dysfunction in older women.

Krüppel-like factors (KLFs), a subfamily of zinc-finger transcription factors, are known to play essential roles in many cellular processes, including proliferation, apoptosis, and differentiation (18). The KLF family plays multiple roles in the regulation of ovarian function, such as KLF2, KLF4, KLF5, KLF9, KLF13, and KLF15 (19, 20, 21, 22). Unlike other members of the KLF family, KLF12 inhibits the transcription of the target gene through the interaction between its amino-terminal PVDLS sequence (Pro-Xaa-Asp-Leu-Ser) and the carboxy-terminal binding protein (23, 24). KLF12 is able to promote apoptosis in a variety of cells, such as endometrial stromal cells (25), ovarian cancer cells (26), and bladder cancer cells (27). A recent study showed that hyperoxia induced KLF2 expression in human lung microvascular endothelial cells, but this was reversed with S1P inhibition (28), suggesting that KLF family molecules might have some correlation regulation with S1P.

In this study, we found that KLF12 expression was significantly upregulated with increasing age in the ovarian GCs of elderly women. A human granulosa cell line (KGN) apoptosis model established by oxidative stress was used to systematically assess the regulatory role of KLF12 in GC apoptosis. In human KGN cell lines, KLF12 expression was elevated with increasing GC apoptosis, accompanied by a decrease in SPHK1 transcriptional activity and S1P production. In contrast, high expression of SPHK1 reversed H_2_O_2_–KLF12-induced apoptosis in human KGN cells. This KLF12–SPHK1–S1P-regulated process was consistent in human KGN/mouse GC (mGC) cells and further fully validated by ovarian *in vitro* culture and 3-nitropropionic acid (3-NP)–induced *in vivo* mouse apoptosis models. Our study reveals the function and mechanism of KLF12 in the ovarian aging process and provides a possible therapeutic target to improve ovarian function in elderly female patients.

## Results

### Increased expression of KLF12 in follicular fluid of aging patients and ovarian GCs under oxidative stress

**Figure 1:**
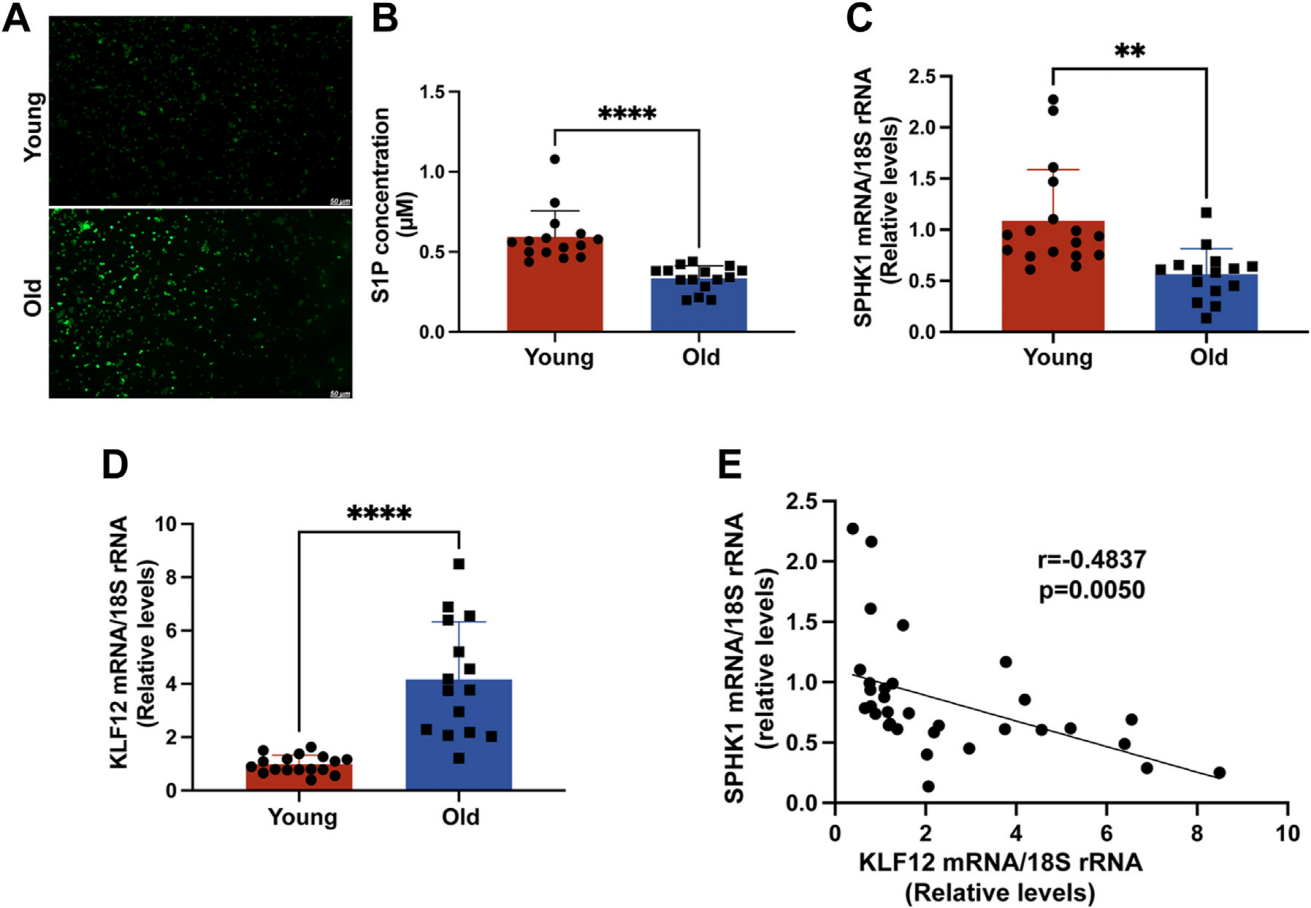
**Increased expression of both reactive oxygen species (ROS) and KLF12 in ovarian GCs of aging patients.***A*, expression levels of ROS in ovarian GCs of young and old patients. The *green color* represents the levels of ROS activity. *B*, S1P expression concentrations in ovarian GCs of young and old patients. The results of each replicate experiment are marked as *circles* and *squares*. *C* and *D*, mRNA expression of *SPHK1/KLF12* in ovarian GCs of young and old patients. *E*, correlation analysis of mRNA expression of *SPHK1* and *KLF12* in human ovarian GCs. The analytical result shows a negative correlation (correlation coefficient *r* = −0.4837) between SPHK1 and KLF12. ∗*p* < 0.05, ∗∗*p* < 0.01, and ∗∗∗*p* < 0.001. GC, granulosa cell; KLF12, Krüppel-like factor 12; S1P, sphingosine-1-phosphate; SPHK1, sphingosine kinase 1.

**Figure 2:**
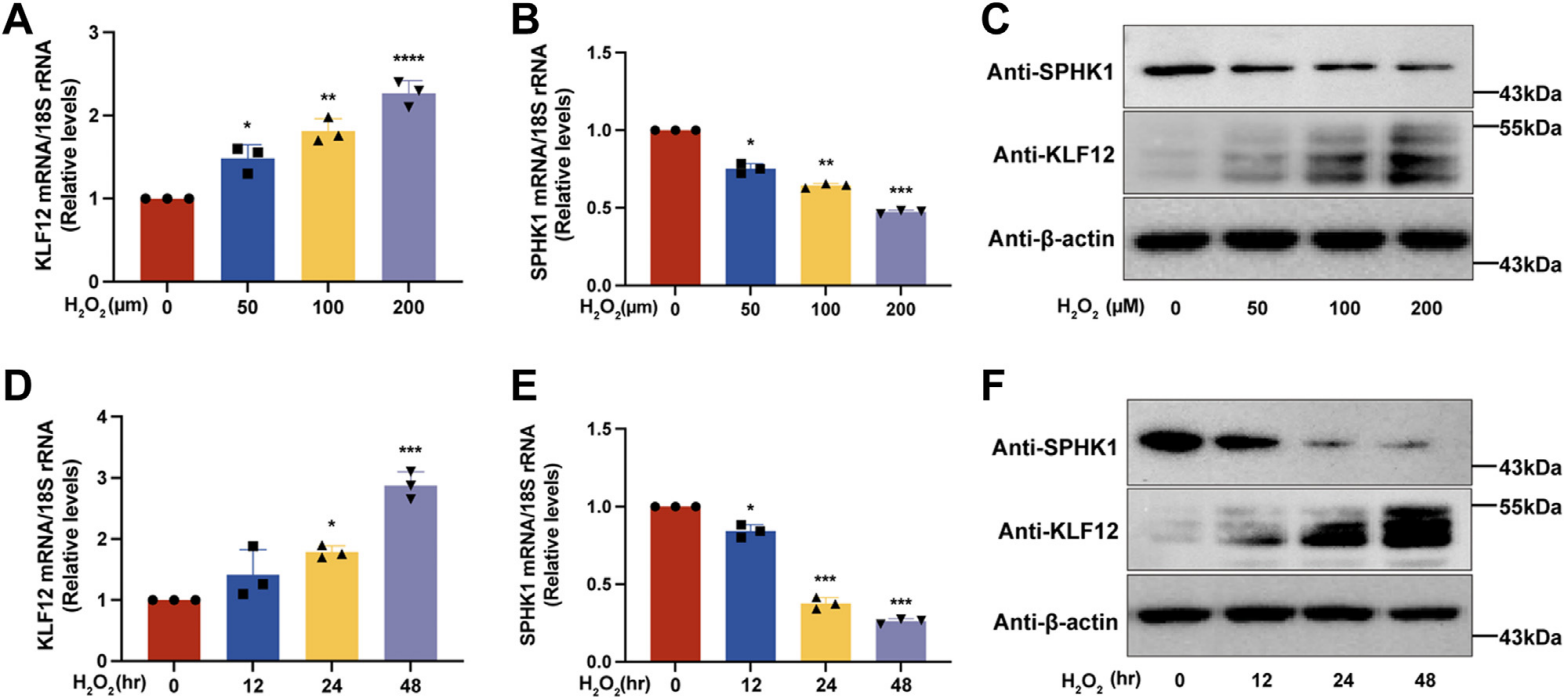
**Dynamic changes of KLF12 in H**_**2**_**O**_**2**_**-induced oxidative stress model in KGN cells.***A* and *B*, changes in mRNA expression of *KLF12/SPHK1* in KGN cells stimulated by different concentrations (0, 50, 100, and 200 μM) of H_2_O_2_. *C*, changes in protein expression of *KLF12/SPHK1* in KGN cells stimulated by different concentrations of H_2_O_2_. Protein expression was normalized to actin levels. *D* and *E*, changes in mRNA expression of *KLF12/SPHK1* in KGN cells under different durations (0, 12, 24, and 48 h) of H_2_O_2_ stimulation. *F*, protein expression changes of KLF12/SPHK1 in KGN cells under different durations of H_2_O_2_ stimulation. Protein expression was normalized to actin levels. ∗*p* < 0.05, ∗∗*p* < 0.01, and ∗∗∗*p* < 0.001. H_2_O_2_, hydrogen peroxide; KLF12, Krüppel-like factor 12; SPHK1, sphingosine kinase 1.

Oxidative stress–induced GC apoptosis is an important mechanism for initiating follicular atresia and is closely associated with the decline in fertility associated with female aging (29, 30). To assess the status of ovarian GCs in patients of different ages, we initially performed an assay for oxidative stress. The excessive increase in ROS suggested the presence of oxidative damage because of oxidative stress in aging GCs (Fig. 1*A*). As a potent antiapoptotic substance, S1P has been reported to protect oocyte quality and embryo development against apoptotic damage from oxidative stress (31, 32). The content of S1P in the GCs of elderly patients with significantly severe oxidative stress was significantly lower than that of young patients (Fig. 1*B*). S1P is generated through phosphorylation of dihydroneurosphingosine by sphingosine kinase (SPHK) (33), and in senescent GCs, a decrease in S1P is accompanied by a decrease in SPHK1 (Fig. 1*C*). However, KLF12 expression was significantly increased in the GCs of aging patients and showed a negative correlation with SPHK1 expression levels (Fig. 1, *D* and *E*). To further investigate the function of KLF12 in human GCs, we stimulated KGN cells with H_2_O_2_ to mimic a state of oxidative stress. The mRNA of KLF12 was significantly upregulated with increasing H_2_O_2_ in a significant concentration-/time-dependent manner (Fig. 2, *A* and *D*). Consistent with the expression in GCs of senescent patients, mRNA expression of SPHK1 also showed a negative correlation with KLF12 expression in KGN cells (Fig. 2, *B* and *E*). We further verified this result at the protein level by Western blotting (Fig. 2, *C* and *F*). These results suggest that the transcription factor KLF12 plays a role in the oxidative stress process in aging ovarian GCs.

### Knockdown of KLF12 delays apoptosis in human ovarian GCs

**Figure 3:**
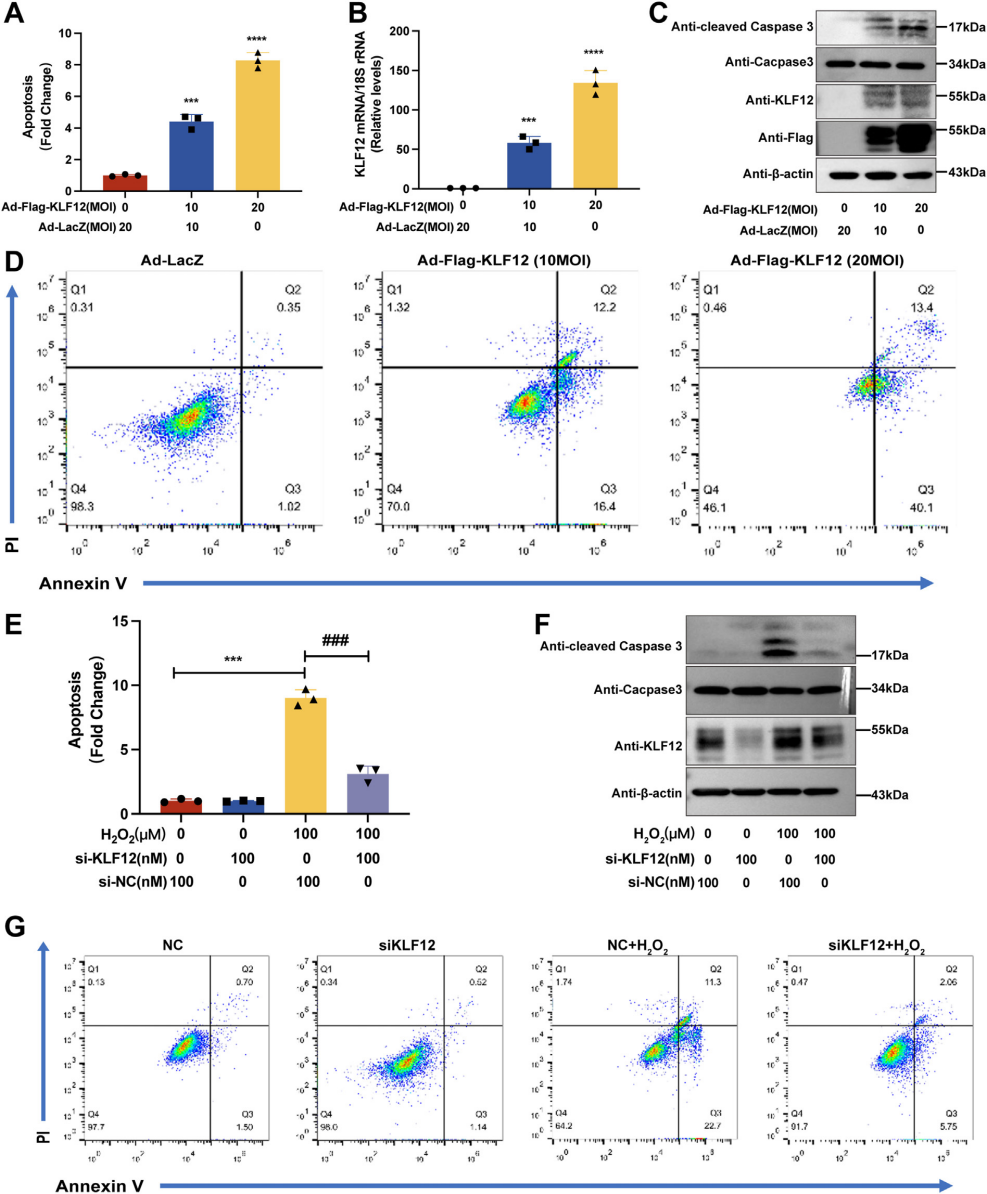
**Knockdown of KLF12 delays H**_**2**_**O**_**2**_**-induced apoptosis in KGN cells.***A*, apoptosis levels in KGN cells with different concentrations of KLF12 overexpression. Adenovirus ad-FLAG-KLF12 was used at 0, 10, and 20 MOI, with corresponding Ad-LacZ at 20, 10, and 0 MOI. *B*, expression levels of *KLF12* in KGN cells with different concentrations of KLF12 overexpression. *C*, detection of apoptosis-related factors in KGN cells with different concentrations of KLF12 overexpression. Caspase-3 was used to detect cell apoptosis. Cleaved caspase-3 was used to assess the degree of cell apoptosis. *D*, flow cytometry analysis of apoptosis in KGN cells with different concentrations of KLF12 overexpression. From *left to right*: Ad-LacZ, ad-FLAG-KLF12 (10 MOI), and ad-FLAG-KLF12 (20 MOI). PI was added for cell analysis. *E*, knockdown of KLF12 delays H_2_O_2_-induced apoptosis in KGN cells. *F*, knockdown of KLF12 delays H_2_O_2_-induced apoptosis-related factor expression in KGN cells. *G*, flow cytometry analysis of the effect of KLF12 knockdown on H_2_O_2_-induced apoptosis in KGN cells. Four groups were set up: NC (no treatment), siKLF12, NC + H_2_O_2_, and siKLF12 + H_2_O_2_. ∗*p* < 0.05, ∗∗*p* < 0.01, and ∗∗∗*p* < 0.001. H_2_O_2_, hydrogen peroxide; KLF12, Krüppel-like factor 12; MOI, multiplicity of infection; PI, propidium iodide.

To investigate the relationship between KLF12 and human GC apoptosis, we observed the effect on apoptosis by overexpressing KLF12 in KGN cells. With the increase in KLF12 expression, the apoptotic signal of KGN cells was significantly enhanced (Fig. 3, *A* and *B*). At the same time, the protein expression of the apoptosis signaling factor anti–cleaved caspase-3 was increased (Fig. 3*C*). The increase in apoptosis was further confirmed by a cell flow assay (Fig. 3*D*). In the KGN cell oxidative stress model, we speculated that knockdown of KLF12 might delay apoptosis. Indeed, in H_2_O_2_-stimulated KGN cells, once KLF12 expression was reduced, the corresponding apoptotic signals were significantly decreased (Fig. 3, *E*–*G*). This result suggests that reducing the expression of KLF12 contributes to delaying apoptosis in ovarian GCs.

### SPHK1 is a novel target gene of KLF12 in human GCs

**Figure 4:**
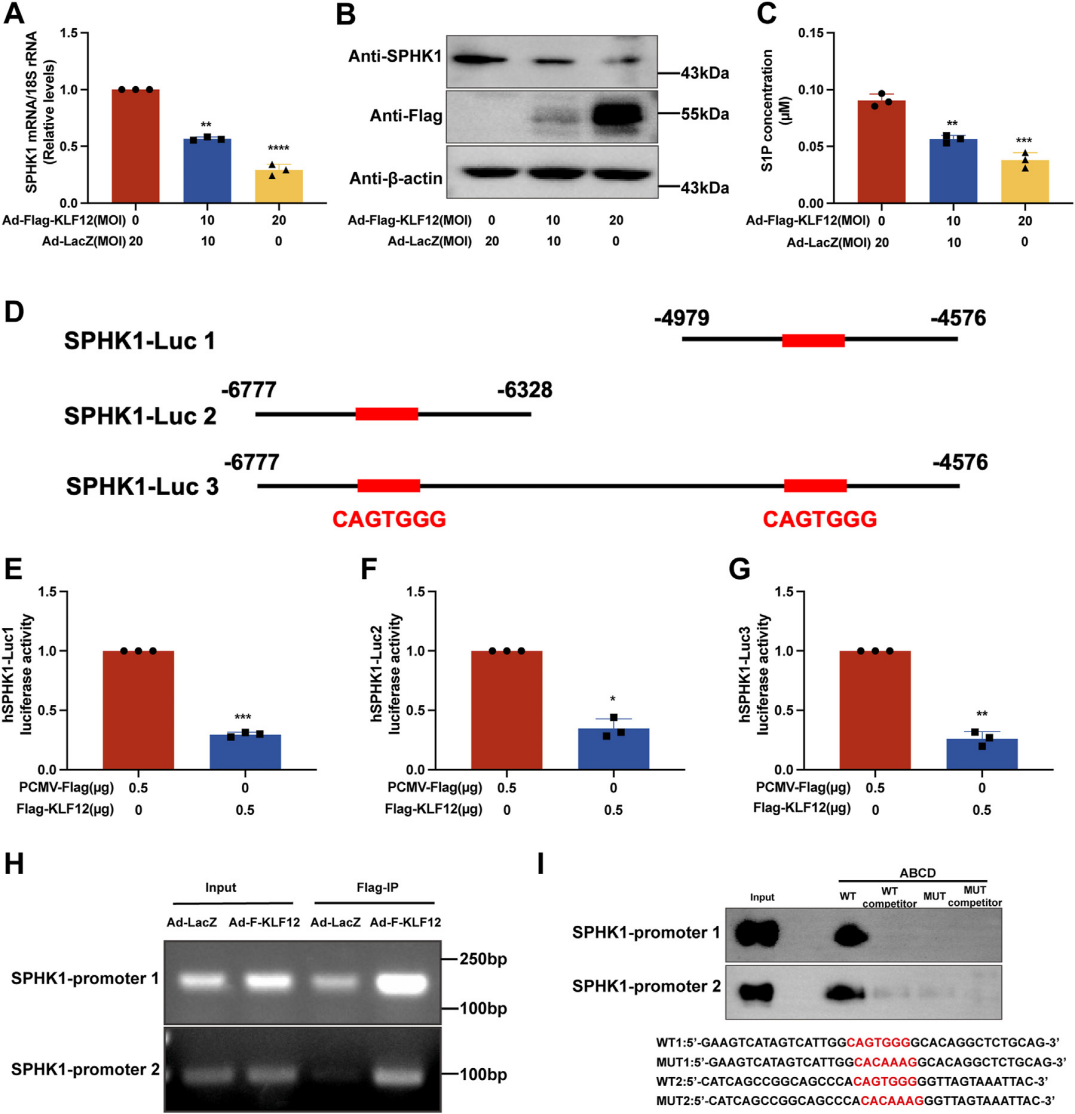
**KLF12 transcriptionally represses SPHK1 expression and reduces S1P production.***A* and *B*, SPHK1 mRNA/protein expression decreases with increasing KLF12 expression. Adenovirus ad-FLAG-KLF12 was applied at 0, 10, and 20 MOI, whereas the corresponding Ad-LacZ was applied at 20, 10, and 0 MOI. *C*, S1P expression concentration decreases with increasing KLF12 expression. *D*, sequence design diagram of SPHK1 promoter (Luc1/Luc2/Luc3). The sequence positions for SPHK1-Luc1 are −4979 to −4576, for SPHK1-Luc2 are −6777 to −6328, and for SPHK1-Luc3 are −6777 to −4576. The *red*-highlighted sequence CAGTGGG is the predicted binding site for KLF12 with the SPHK1 promoter. *E*–*G*, luciferase reporter assay of SPHK1 promoter (Luc1/Luc2/Luc3). When FLAG-KLF12 is expressed in the KGN cell line, SPHK1 luciferase activity significantly decreases by more than half. *H*, chromatin immunoprecipitation (ChIP) assay of SPHK1 promoter (Luc1/Luc2) and KLF12. KLF12 displays strong binding efficiency with the SPHK1 promoter (Luc1/Luc2). *I*, avidin–biotin conjugate DNA (ABCD) precipitation assay for SPHK1 of WT and MUT. KGN cells transfected with Ad-FLAG-KLF12 were lysed, and the binding ability between DNA lysate products and SPHK1-WT1, SPHK1-WT2, SPHK1-MUT1, and SPHK1-MUT2 was compared. Results show that the DNA lysate products rich in KLF12 have a strong binding ability with SPHK1-WT. ∗*p* < 0.05, ∗∗*p* < 0.01, and ∗∗∗*p* < 0.001. KLF12, Krüppel-like factor 12; MOI, multiplicity of infection; MUT, mutated probe; S1P, sphingosine-1-phosphate; SPHK1, sphingosine kinase 1.

Based on the dynamic changes in KLF12 and SPHK1 in human aging ovarian GCs, we hypothesized that KLF12 had some negative associations with SPHK1. Indeed, after the overexpression of different concentrations of KLF12 in KGN cells, the SPHK1 concentration showed a decreasing trend at the mRNA/protein level (Fig. 4, *A* and *B*). Similarly, S1P levels also decreased with increased expression of KLF12 (Fig. 4*C*). Interestingly, bioinformatics analysis revealed that the promoter region of the human SPHK1 gene has multiple KLF12 protein–conserved binding elements (CAGTGGG), which suggests that SPHK1 could be a novel target of KLF12.

**Supporting Figure S1:**
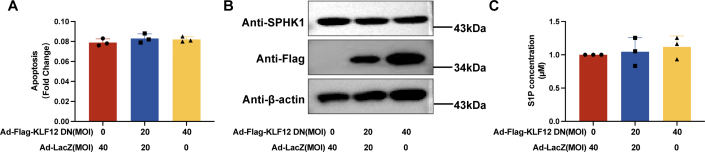
**High expression of Ad-KLF12 DN does not affect the apoptosis of KGN cells.***A*, investigating the effect of Ad-KLF12 DN, a high-expression adenovirus of KLF12 with functional structure deletion, on apoptosis. *B* and *C*, assessing the impact of high expression of Ad-KLF12 DN on the protein expression of SPHK1 and S1P production, which was found to be non-significant. The overexpression of Ad-KLF12 DN in KGN cells did not affect apoptosis, thus ruling out any non-specific effects of KLF12 overexpression. Statistical significance: ∗*P* < 0.05, ∗∗*P* < 0.01, ∗∗∗*P* < 0.001.

To localize the KLF12-binding site within this promoter, we first generated three SPHK1-Luc reporter constructs (SPHK1-Luc1/2/3) (Fig. 4*D*), which were used in luciferase reporter assays. Second, the luciferase reporter assay demonstrated that SPHK1 promoter activity could be repressed by KLF12 overexpression in KGN cells (Fig. 4, *E*–*G*). In addition, conventional chromatin immunoprecipitation (ChIP)–PCR analysis was conducted and revealed that both promoters (−4979 to −4576 bp, −6777 to −6328 bp) were effectively recovered from immunoprecipitants of the FLAG-KLF12 protein, but they were not recovered from those of the LacZ control (Fig. 4*H*). Finally, ABCD assays were performed using biotinylated double-stranded oligonucleotides corresponding to the WT and mutated (MUT) SPHK1 promoter sequences, and the results showed that the FLAG-tagged KLF12 proteins strongly bound to the WT probe but not to the MUT probe (Fig. 4*I*). Moreover, to further explore the function of KLF12 in ovarian GCs and confirm the relationship between KLF12 and SPHK1, an adenovirus overexpressing KLF12 with zinc finger structure deletion (Ad-FLAG-KLF12 DN), in which KLF12 has no transcriptional activity, was employed to infect GCs, and we found that KLF12 without its zinc finger structures could not inhibit the expression of SPHK1 and had no effect on the apoptosis of ovarian GCs (Fig. S1). Together, these results suggest that SPHK1 is transcriptionally regulated by KLF12.

### Supplementation of SPHK1 partially rescues H_2_O_2_–KLF12-induced apoptosis in human/mouse ovarian GCs

**Figure 5:**
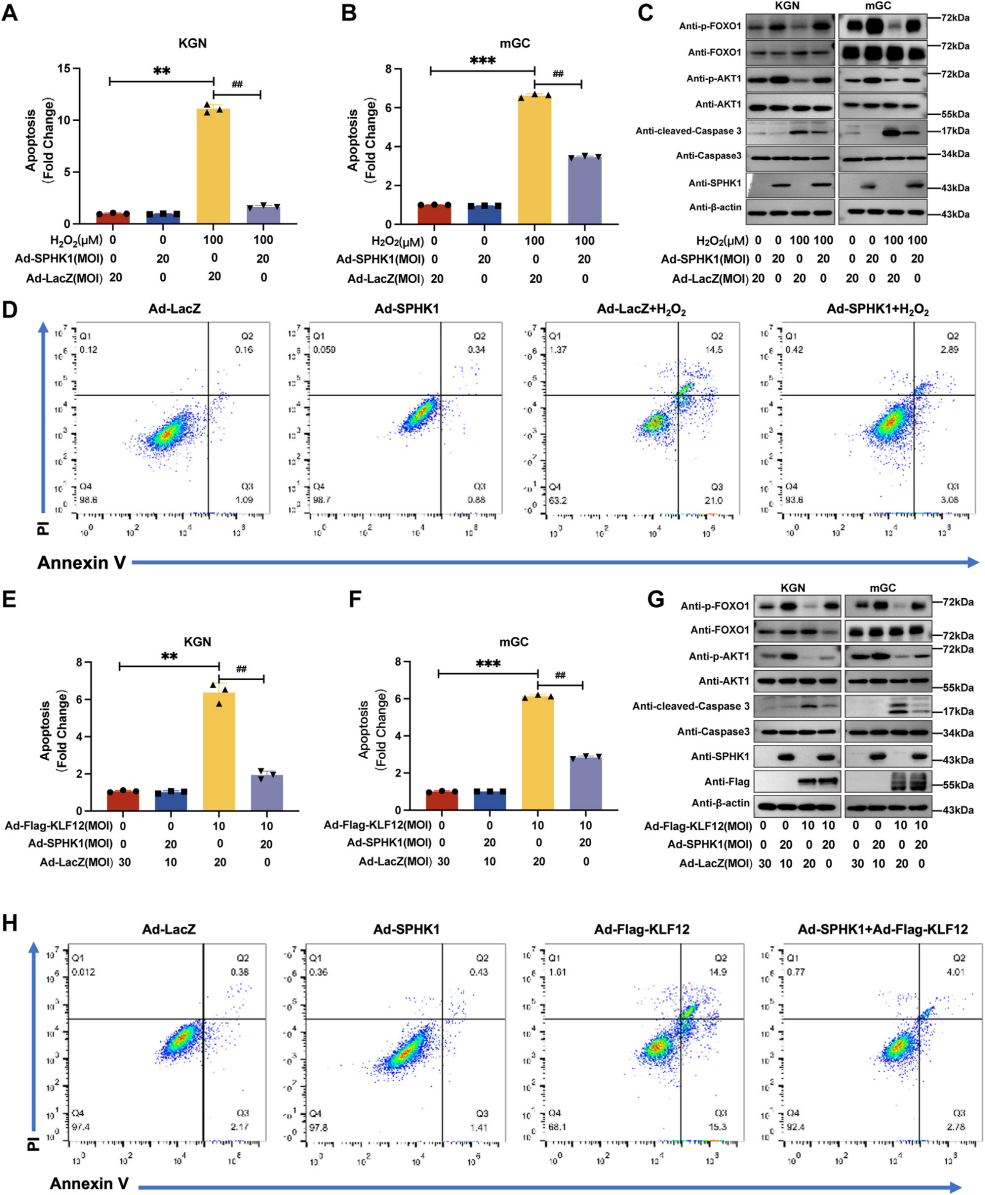
**The overexpression of SPHK1 partially rescues H**_**2**_**O**_**2**_**or KLF12-induced apoptosis in KGN and mGC.***A*–*C*, the expression levels of various apoptotic markers were evaluated to unravel the underlying mechanism. Treatment with H_2_O_2_ or KLF12 increased cleaved caspase-3 expression levels, indicating augmented apoptosis. However, the overexpression of SPHK1 significantly attenuated the apoptosis levels (with increased expression levels of p-FOXO1 and p-AKT1). *D*, for cell flow cytometry analysis, propidium iodide (PI) was added, and different types of apoptotic cells were distinguished based on their Annexin V and PI labeling. *E*–*G*, the overexpression of SPHK1 partly rescued KLF12-induced apoptosis in KGN/mGC cells, which was associated with increased expression levels of p-FOXO1 and p-AKT1. *H*, a cell flow cytometry plot was used to evaluate KLF12 rescue of H_2_O_2_-induced apoptosis in KGN cells. ∗*p* < 0.05, ∗∗*p* < 0.01, and ∗∗∗*p* < 0.001. H_2_O_2_, hydrogen peroxide; KLF12, Krüppel-like factor 12; mGC, mouse granulosa cell; SPHK1, sphingosine kinase 1.

As SPHK1–S1P has a protective effect on cell survival and SPHK1 was determined to be a novel target of KLF12, we further investigated whether SPHK1–S1P was able to reverse the induced apoptosis in GCs stimulated by KLF12 overexpression or H_2_O_2_. KLF12-enhanced mGCs were treated with adenovirus-mediated overexpression of SPHK1 to determine the complementary role. SPHK1 overexpression reversed H_2_O_2_–KLF12-induced apoptosis in nearly half of mGCs and human KGN cells (Fig. 5, *A*, *B*, *E* and *F*).

**Supporting Figure S2:**
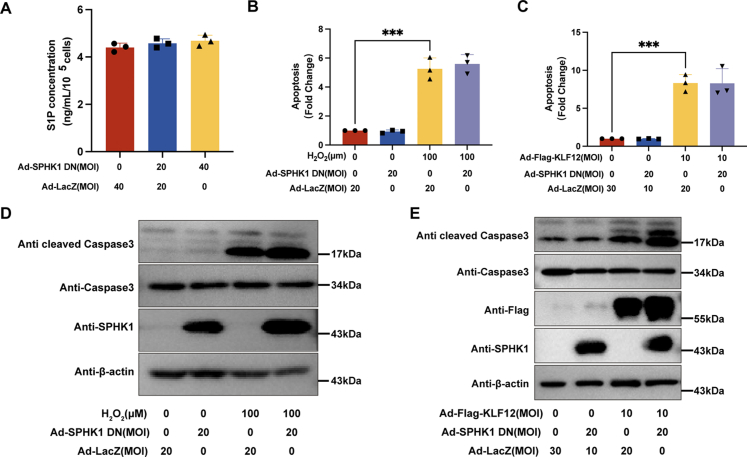
**High expression of Ad-SPHK1 DN cannot rescue apoptosis of KGN cells.***A*, investigating the effect of high expression of Ad-SPHK1 DN on S1P concentration. *B* and *C*, examining the impact of high expression of Ad-SPHK1 DN on H_2_O_2_/KLF12-induced apoptosis, which was found to be non-rescuable. *D* and *E*, analyzing changes in apoptotic factors in H_2_O_2_/KLF12-induced KGN cells by high expression of Ad-SPHK1 DN. The overexpression of Ad-SPHK1 DN in KGN cells did not affect apoptosis, thus ruling out any non-specific effects of SPHK1 overexpression. Ad-SPHK1 DN is a high-expression adenovirus of SPHK1 with functional structure deletion. Statistical significance: ∗*P* < 0.05, ∗∗*P* < 0.01, ∗∗∗*P* < 0.001.

Concurrently, in H_2_O_2_-stimulated or KLF12-overexpressing mGCs or KGN cells, the total contents of AKT and FOXO1 did not change significantly, whereas their phosphorylation levels, which have been reported to inhibit apoptosis, were significantly reduced. The important apoptotic molecule cleaved caspase-3 was significantly upregulated, although the total amount of caspase 3 was unchanged. However, SPHK1 overexpression in H_2_O_2_-stimulated or KLF12-overexpressing mGCs or KGN cells significantly reversed AKT and FOXO1 phosphorylation levels and suppressed cleaved caspase-3 expression (Fig. 5, *C*, *D*, *G* and *H*). Simultaneously, a high-expression adenovirus of SPHK1 with functional structure deletion (Ad-SPHK1 DN), where SPHK1 cannot generate S1P, was employed in compensation experiments, and we found that Ad-SPHK1 DN could not promote S1P production and reverse KLF12-induced apoptosis of KGN cells (Fig. S2). Together, the functional structure of SPHK1 plays a critical role in promoting S1P synthesis and its antiapoptotic effect.

### Supplementation with SPHK1 partially rescues 3-NP/KLF12-induced apoptosis in ovarian GCs in an ovarian culture model

**Figure 6:**
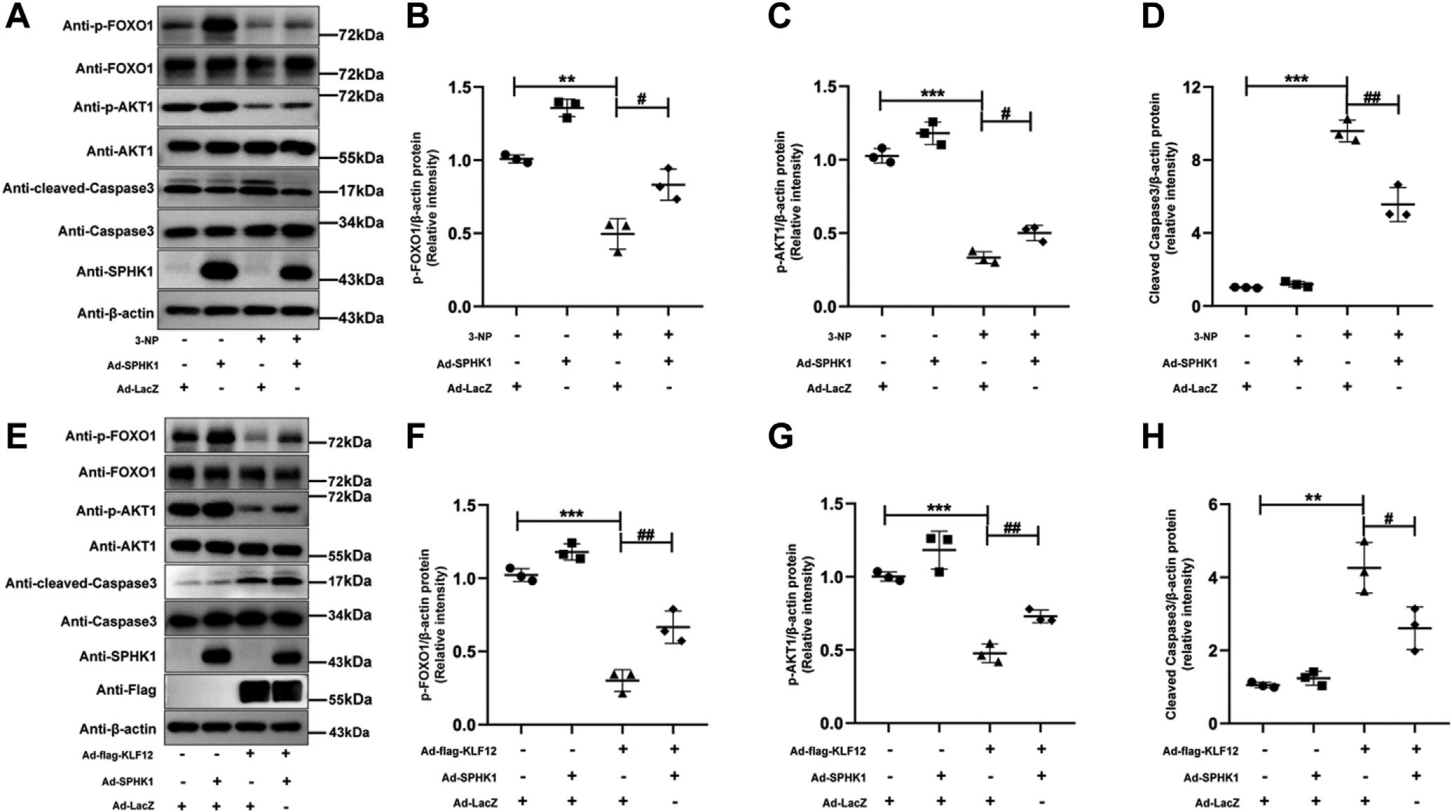
**SPHK1 supplementation partially rescues 3-NP/KLF12-induced apoptosis in ovarian GCs in an ovarian culture model.***A*, SPHK1 supplementation mitigates 3-NP-induced apoptosis-related factors as analyzed through protein assays in an ovarian culture model. *B*–*D*, p-FOXO1, p-AKT1, and cleaved caspase-3 expression levels indicate changes in the 3-NP-induced ovarian culture model. 3-NP treatment increases apoptosis in ovarian GCs (elevated cleaved caspase-3 expression), but SPHK1 expression significantly counteracts the increase in apoptosis levels (increased p-FOXO1 and p-AKT1 expression levels). *E*, SPHK1 supplementation mitigates KLF12-induced apoptosis-related factors as detected through protein assays in an ovarian culture model. *F*–*H*, p-FOXO1, p-AKT1, and cleaved caspase-3 expression levels indicate changes in the KLF12-induced ovarian culture model. Overexpression of KLF12 increases apoptosis in ovarian GCs (elevated cleaved caspase-3 expression), but SPHK1 expression significantly counteracts the increase in apoptosis levels (increased p-FOXO1 and p-AKT1 expression levels). ∗*p* < 0.05, ∗∗*p* < 0.01, and ∗∗∗*p* < 0.001. 3-NP, 3-nitropropionic acid; GC, granulosa cell; KLF12, Krüppel-like factor 12; SPHK1, sphingosine kinase 1.

To validate the role of KLF12-induced GC apoptosis in the whole ovary, oxidative stress–induced GC apoptosis was established in *in vitro*–cultured ovaries. 3-NP, a well-known toxin of *Indigofera*, may cause oxidative stress (34, 35). Consistent with the validation in GCs, 3-NP or KLF12 overexpression induced a significant decrease in the phosphorylation levels of AKT and FOXO1 and a significant increase in cleaved caspase-3 expression in mouse whole ovaries, although the total contents of AKT and FOXO1, as well as caspase 3 expression, did not change significantly. Nevertheless, SPHK1 overexpression in 3-NP-stimulated or KLF12-overexpressing mouse whole ovaries significantly reversed AKT and FOXO1 phosphorylation levels and suppressed cleaved caspase-3 expression (Fig. 6). Together with the results of KGN cells and mGCs in the *in vitro*–cultured ovary, these results demonstrate that KLF12 induces functional ovarian apoptosis by decreasing the expression of SPHK1–S1P.

### SPHK1 antagonizes the expression of KLF12 in an ovarian oxidative stress model *in vivo*

**Figure 7:**
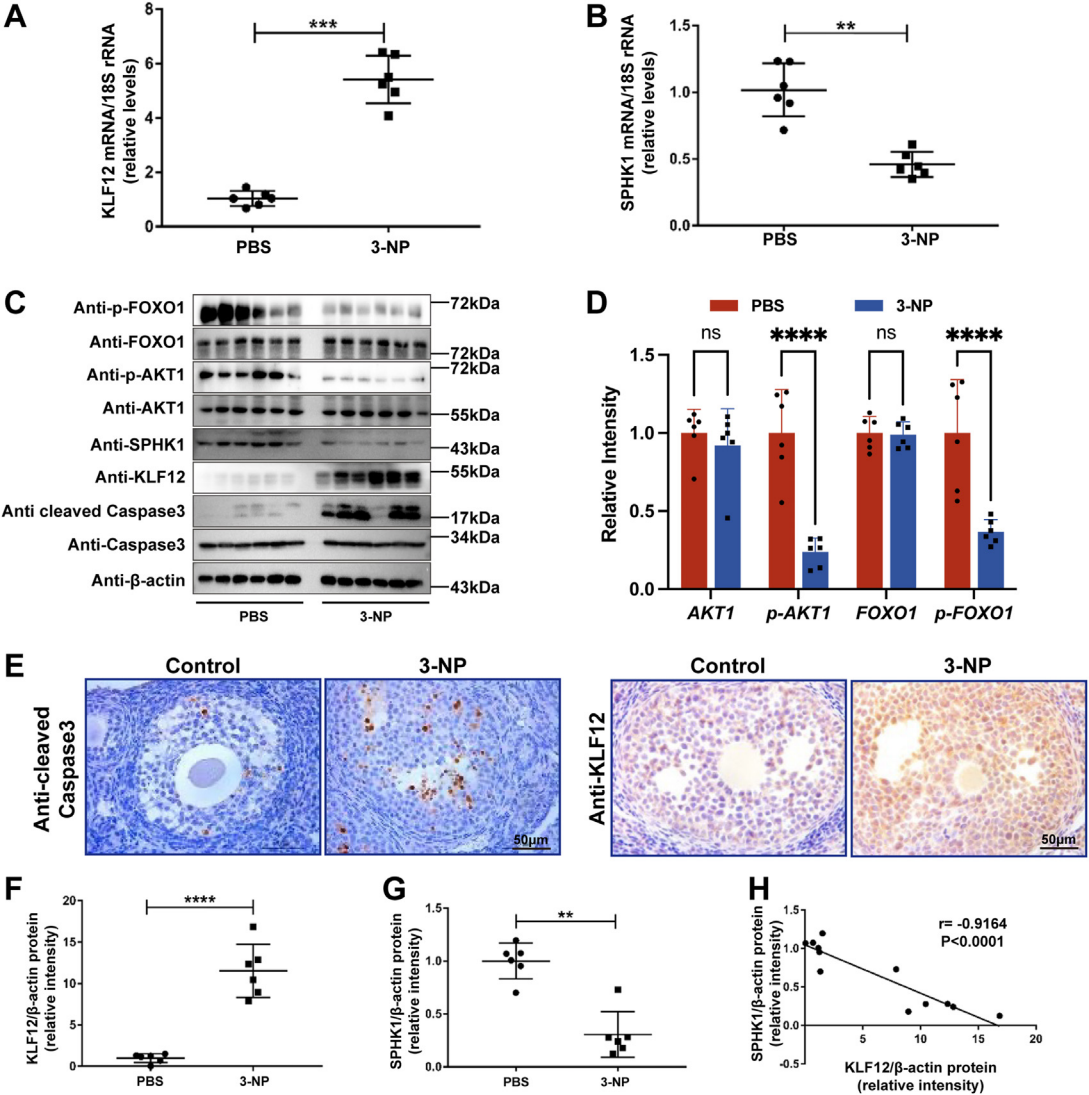
**KLF12 and SPHK1 exhibit negative correlation in 3-NP-induced *in vivo* mouse ovaries.***A* and *B*, mRNA expression of KLF12 and SPHK1 in 3-NP-induced *in vivo* mouse ovaries. *C*–*F*, protein expression of KLF12, SPHK1, and cleaved caspase-3 in 3-NP-induced *in vivo* mouse ovaries. In 3-NP-induced mouse ovaries, apoptotic factor (cleaved caspase-3) expression increases and anti-apoptotic factors (p-FOXO1 and p-AKT1) expression decreases. KLF12 expression significantly increases, and SPHK1 expression significantly decreases, presenting a negative correlation. *G*, KLF12 and SPHK1 show a negative correlation in 3-NP-induced *in vivo* mouse ovaries. *H*, immunohistochemistry reveals increased expression of cleaved caspase-3 and KLF12 in 3-NP-induced oxidative stress ovaries. The scale bar represents 50 μm. ∗*p* < 0.05, ∗∗*p* < 0.01, and ∗∗∗*p* < 0.001. 3-NP, 3-nitropropionic acid; KLF12, Krüppel-like factor 12; SPHK1, sphingosine kinase 1.

To study GC apoptosis induced by oxidative stress more effectively, we established a 3-NP-induced ovarian oxidative stress mouse model. In the oxidative stress mice, cleaved caspase-3 and KLF12 expression was significantly higher than that in the control mice, whereas there was a significant decrease in SPHK1 expression in the oxidative stress model mice compared with the controls (Fig. 7, *A*–*G*). Furthermore, we found a negative correlation between KLF12 and SPHK1 expression (Fig. 7*H*). Combined with *in vitro*–cultured ovaries and *in vivo* 3-NP-induced ovarian oxidative stress mice, it is suggested that oxidative stress–induced ovarian GC apoptosis may be regulated by KLF12–SPHK1–S1P.

## Discussion

Oxidative stress induced by factors such as aging can increase GC apoptosis and accelerate the atresia of follicles or the early depletion of oocytes, leading to ovarian function decline or ovarian aging. KLF12 was reported to be a novel apoptosis-inducing molecule that plays an important negative role in reproductive function (25, 26), and our previous work confirmed that KLF12 significantly increased in ovarian GCs after oxidative stress stimulation; however, little is known about the detailed function and mechanism in ovarian GCs. Herein, KLF12, a novel molecule regulating ovarian GC apoptosis, was identified for the first time. Abnormally enhanced KLF12 expression promoting GC apoptosis by transcriptional inhibition of SPHK1 expression was demonstrated.

Kruppel-like factors (KLFs), a subfamily of zinc finger transcription factors, have been reported to be involved in the treatment of antiaging and apoptosis or autophagy-related diseases. A recent study showed that KLF5 collaborated with FOXO1 to regulate atrophy-related processes, including metabolic changes and E3-ubiquitin ligase–mediated proteolysis, and KLF5 expression increased significantly with age and sarcopenia and was positively correlated with the expression of the atrophy-related ubiquitin ligase genes *FBXO32* and *TRIM63* (36). KLF1 or KLF3 prolongs the life span of *Caenorhabditis elegans* and delays the appearance of age-related phenotypes, such as decreased locomotion speed, by regulating autophagy (37). In mammals, the regulation of autophagy by KLFs, including KLF2, KLF4 and KLF6, is also involved in vascular aging, heart failure, and cell aging (37, 38). The defining feature of the KLF family is the carboxy-terminal DNA-binding domain, which consists of three zinc fingers that bind GC-rich DNA sequences. The rest of the amino acid sequence is divergent, and each KLF typically contains at least one transactivation or transrepression domain. Distinctively, KLF12 possesses an N-terminal repression domain that contains a carboxy-terminal binding protein recognition motif (39, 40, 41). All these findings suggest that KLFs are potential therapeutic targets for diseases such as antiaging and apoptosis or autophagy-related diseases.

Interestingly, our previous research has demonstrated that an increase in miR-181a expression can also promote GC apoptosis (42). Furthermore, downregulation of miR-181a can significantly enhance S1P receptor 1 expression (43). These findings, coupled with the phenotype of KLF12-promoted cell apoptosis, suggest that KLF12 and miR-181a may be coregulating apoptosis of GCs in a synergistic manner.

In addition to SPHK1, there is another member called SPHK2, which is an important rate-limiting enzyme responsible for maintaining the balance of sphingolipids. Synergistically, SPHK2 in the cytoplasm has a similar function as SPHK1, which can catalyze sphingosine to generate S1P (44). Therefore, we also analyzed the promoter sequence of *SPHK2* and found that it also contained the conserved binding site of KLF12. However, in ovarian GCs, abnormally enhanced KLF12 expression had no significant effect on the *SPHK2* protein and mRNA expression levels (data not shown), which suggested that KLF12 could promote GC apoptosis by inhibiting the expression of SPHK1 to affect the synthesis of S1P.

S1P has long been considered an inhibitor of apoptosis. A study reported that S1P can regulate and stimulate cell growth and antagonize ceramide-induced apoptosis. Early studies have confirmed that S1P can significantly protect GCs from apoptosis triggered by oxidative stress, which is induced by chemotherapy, radiotherapy, or H_2_O_2_, by activating PI3K–Akt signaling (12, 13, 14). A recent study demonstrated that an oral analog of S1P might inhibit spontaneous apoptosis of age-related follicular cells and could increase the ratio of nonapoptotic follicles and anti-Mullerian hormone levels (45). In our study, we found that the content of S1P in the GCs of older patients was significantly lower than that of young patients. Moreover, the expression of S1P in the supernatant of ovarian GCs induced by H_2_O_2_ was significantly repressed, suggesting that S1P plays an important role in maintaining female ovarian function. Similar to our results, a relevant study has shown that S1P is elevated in GCs of healthy developing follicles and significantly reduced in GCs of atretic follicles (46). It is well known that S1P is formed by SPHK catalyzing sphingosine phosphorylation (47, 48). Studies have shown that SPHK1–S1P can protect cell survival by activating the PI3K–Akt–FOXO or extracellular signal–regulated kinase pathway and inhibit apoptosis induced by oxidative stress or chemotherapy drugs (49, 50). SPHK1–S1P is regulated by bone morphogenetic protein 2, protecting bovine GC proliferation and gonadotropin-independent folliculogenesis through Hippo pathway suppression (51). These results suggest that SPHK1–S1P plays an important role in regulating the microenvironment of follicular development and reveal the upstream molecules of SPHK1–S1P. In our study, KLF12 was also identified as an important transcription factor upstream of SPHK1–S1P; however, it has a different role, that is, promoting GC apoptosis and follicular atresia. In addition, the biological function of S1P agonists or S1PR antagonists in the regulation of GC apoptosis in the 3-NP mouse model and KLF12 conditional knockout mouse should be further investigated, and these results may be beneficial for developing novel clinical therapies, which can alter the local microenvironment of the ovary.

In conclusion, our study revealed a new mechanism by which KLF12 regulates GC apoptosis in the ovarian aging process and provided a new possibility for the clinical treatment of premature ovarian failure to identify targets.

## Experimental procedures

### Patients and sample collection

All participants gave their fully informed consent before undergoing *in vitro* fertilization treatment. Human GCs were separated from two groups of participants of different ages: the younger group was younger than 29 years (n = 17, range, 22–30 years), and the older group was older than 37 years (n = 15, range, 37–46 years). Ethical approval for this study was obtained from the Drum Tower Hospital of Nanjing University Medical School (2013-081-01). The studies in this work abide by the Declaration of Helsinki principles.

### Mice

The animal experiments in this study were approved by the Institutional Animal Care and Use Committee of Nanjing Drum Tower Hospital (SYXK 2019-0059). Three-week-old female Institute of Cancer Research (ICR) mice were purchased from the Nanjing Medical University Animal Center and then housed at the Nanjing Drum Tower Hospital Animal Center on a 12/12 h light/dark cycle.

### Isolation of primary mGCs and cell lines

The ovaries of 3-week-old female ICR mice were isolated to acquire primary mGCs by puncture with a 25-gauge needle. Oocytes were removed using a 40 μm cell filter, and mGCs were purified. The mGCs were cultured in Dulbecco's modified Eagle's medium/F12 medium (Corning) supplemented with 10% fetal bovine serum (Corning), 1 mM sodium pyruvate (HyClone), 2 mM glutamine (HyClone), 100 U/ml penicillin, and 100 μg/ml streptomycin (HyClone). KGN cells (human ovarian GC line) were a gift from Professor Hu (Nanjing Drum Tower Hospital) and grown in Dulbecco's modified Eagle's medium/F12 medium supplemented with 100 μg/ml streptomycin, 100 U/ml penicillin, and 10% (v/v) bovine serum (bovine calf serum; Sigma‒Aldrich). All cells were maintained at 37 °C with 5% CO_2_. In some experiments, mGCs or KGN cells were starved overnight at 90% density and then treated with 100 μM H_2_O_2_ (Sigma‒Aldrich) for 0, 24, 48, or 72 h.

### *In situ* hybridization

Mouse ovarian tissue was fixed using 4% paraformaldehyde for 20 min at room temperature and cut into 10 μm sections. Sections were subsequently treated with 0.25% and 0.5% acetic anhydride for 5 min. The DIG-*Klf12* probe was then preheated at 60° and covered with silanized glass overnight. The slides were washed twice using hybrid wash solution at 60 °C for 30 min and subsequently cooled to room temperature. The sections were washed with MABT solution for 30 min at room temperature and RNA solution for 10 min at 37 °C. Pretreat with RNA solution (10 mg/ml RNase) at 37 °C for 30 min, followed by switching to fresh RNA solution at 37 °C for 5 min, and then to MABT solution at room temperature for 5 min. After blocking, sections were incubated with antidigoxin antibodies (1:200 dilution; Roche) overnight at 4°. After washing once with MABT solution and NTM solution, sections were treated with levamisole (0.5 mg/ml) for 10 min. According to the manufacturer's instructions, staining signals were visualized using 5-bromo-4-chloro-3-indolyl-phosphate/nitroblue tetrazolium (Beyotime Biotechnology) as a substrate. Subsequently, sections were stained with 0.1% Nuclear Fast Red solution (Phygene) and sealed.

### Generation of recombinant adenovirus

Adenovirus ad-FLAG-KLF12, ad-FLAG-SPHK1, and other highly expressed adenoviruses were constructed by the AdMax system (Microbix). Ad-lacZ was purchased from Clontech. After amplification in 293A cells, the adenovirus was purified by cesium chloride and stored in 20 mM Tris buffer containing 10% glycerol at −70 °C. The adenovirus titer was determined according to the instructions of the adeno-X Rapid Titer (Clontech) kit.

### siRNA synthesis and transfection

KLF12 siRNA and negative control siRNA were synthesized by Guangzhou RiboBio Co, Ltd. KLF12 siRNA sequences were as follows: siKLF12-forward: 5′-GCAAUCGAAUGAAUAAUCAUU-3′ and siKLF12-reverse: 5′-UGAUUAUUCAUUCGAUUGCUU-3′. Prior to transfection, mGCs or KGN cells (1 × 10^5^ cell/well) were plated in a 6-well plate with complete medium and incubated overnight. KLF12 or negative control siRNA was transfected into mGCs or KGN cells with Lipofectamine 2000 Transfection Reagent (Thermo Fisher Scientific). The specific transfection scheme was performed according to the reagent manufacturer's instructions. After 48 h, the transfected mGCs or KGN cells were used for further experiments.

### RNA isolation and quantitative real-time PCR

Total RNA in GCs was extracted using TRIzol Reagent (Invitrogen) and subsequently reverse transcribed into complementary DNA. Reverse transcription was performed using random primers, and RT‒quantitative PCR was performed using the LightCycler 480 Instrument. All experiments were repeated three times. The list of primers is shown in Table S1.

### Western blot assay

Proteins were extracted from cultured GCs or ovarian tissues as described previously (52). Briefly, protein concentrations were measured using BCA Protein Assay Reagent (Thermo Fisher Scientific). Proteins were separated using SDS-PAGE and transferred to polyvinylidene fluoride membranes (Millipore). After incubation with primary antibodies and secondary antibodies of the corresponding species, exposures were recorded using a chemiluminescent horseradish peroxidase substrate kit (Millipore). The following antibodies were used: β-actin (1:10,000 dilution, catalog no.: AP0060; Bioworld Technology), KLF12 (1:500 dilution, catalog no.: sc-134373; Santa Cruz Biotechnology), SPHK1 (1:500 dilution, catalog no.: CY6962; Abways), cleaved caspase-3 (1:500 dilution, catalog no.: 9661S; Cell Signaling Technology), caspase-3 (1:1000 dilution, catalog no.: 9662S; Cell Signaling Technology), p-AKT (1:500 dilution, catalog no.: sc135650; Santa Cruz Biotechnology), and AKT (1:500 dilution, catalog no.: BS2987; Bioworld Technology), p-FOXO1(1:500 dilution, catalog no.: ab47326; abcam), and FOXO1 (1:2000 dilution, catalog no.: 2880S; Cell Signaling Technology).

### Cell death detection assay

Apoptosis was detected using a cell death detection ELISA kit (Roche). Briefly, cells were washed twice with prechilled PBS and gently shaken with lysis buffer for 30 min at room temperature. About 20 μl of supernatant was taken after centrifugation at 250*g* for 10 min. Finally, the relative cell death was determined by reading the absorbance at 405 nm/490 nm with a 96-well plate.

### Cell apoptosis detection by flow cytometry

To determine the effects of H_2_O_2_–KLF12 on the induction of apoptosis in GCs, the TACS Annexin V-FITC Apoptosis Detection Kit (R&D Systems, Inc) was used according to the manufacturer’s guidelines. Briefly, GCs were digested with trypsin–EDTA. The cells were collected, gently washed with PBS, and counted. Cells were collected by centrifugation at approximately 300*g* for 5 min at room temperature and resuspended in Annexin V Incubation Reagent at a concentration of 1 × 10^6^ cells/100 μl. Cells were incubated in the dark for 15 min at room temperature. Finally, 400 μl of 1× binding buffer was added to stained cells and acquired by Navios flow cytometry (Beckman Coulter Life Science) within 1 h for maximal signal. The results were analyzed by Navios software (Beckman). Finally, 10 μl propidium iodide (PI) was added for cell flow cytometry analysis. Viable cells (Annexin V^−^/PI^−^) do not take any color, early apoptotic cells (Annexin V^+^/PI^−^) are *green*, late apoptotic cells (Annexin V^+^/PI^+^) are *green* and *orange*, and necrotic cells (Annexin V^−^/PI^+^) are *orange*.

### Luciferase reporter assay

293_cells were transfected with pGL3-basic luciferase reporter plasmids loaded with the SPHK1 promoter (Luc1/Luc2/Luc3) when they reached 70% confluence using Lipo2000 transfection reagent. Forty-eight hours later, the cells were collected, and luciferase activity was subsequently measured using a dual luciferase assay system (Promega) The luciferase activity data were measured using a luminescence counter (Berthold Technologies). Firefly luciferase activity was standardized according to Renilla luciferase activity.

### ChIP assay

KGN cells were infected with Ad-LacZ and Ad-FLAG-KLF12 at a growth density of 60%, and the multiplicity of infection was set at 20. KGN cells were collected for ChIP after 48 h. The recovered DNA using FLAG beads was analyzed by PCR. The specific primer sequences used for ChIP–PCR are listed below. *SPHK1*-F1 5′-ATGAAAAATTCCAGAGCAGTGAGTG-3′ and *SPHK1*-R1 5′-GCTATCCCTTAAGAAAAGCTGCTTT-3′, *SPHK1*-F2 5′-CATCGCCATGGTAAGGAGCT-3′, and *SPHK1*-R2 5′-CATCCAAGAAAACAGCATAT-3′.

### Avidin–biotin conjugate DNA precipitation assay

Double-stranded oligonucleotides were designed based on the human *SPHK1* (gene ID: 8877) promoter sequence. The 5′ end of the sense strand was biotinylated, and a deletion and a mutation were introduced (mutation of the CAGTGGG sequence) to remove the specific binding site for *KLF12*. The following primers were designed: *SPHK1* WT1: 5′-GAAGTCATAGTCATTGGCAGTGGGGCACAGGCTCTGCAG-3′; *SPHK1* WT2: 5′-CATCAGCCGGCAGCCCACAGTGGGGGTTAGTAAATTAC-3′; *SPHK1* MUT1: 5′-GAAGTCATAGTCATTGGCACAAAGGCACAGGCTCTGCAG-3′, and *SPHK1* MUT2: 5′-CATCAGCCGGCAGCCCACACAAAGGGTTAGTAAATTAC-3′. KGN cells infected with Ad-LacZ and Ad-FLAG-KLF12 (multiplicity of infection = 20) for 48 h were harvested and lysed. Each double-stranded DNA sample (500 pmol) was incubated with 600 μg of cell extract at 4 °C for 4 h, and the protein complexes were pulled down using streptavidin agarose beads (Sigma) in binding buffer (10 mM Tris, pH 8.0, 0.5% Triton X-100, 150 mM NaCl, 10% glycerol, 0.5 mM EDTA, 0.5 mM DTT, and 20 μg/ml poly [dI–dC]) containing a protease inhibitor cocktail. The beads were washed four times with the same buffer to obtain the bound proteins. The obtained proteins were separated by SDS-PAGE electrophoresis and then transferred to a polyvinylidene fluoride membrane. Protein expression signals were detected using enhanced chemiluminescence (Millipore). The probes were detected with a FLAG-horseradish peroxidase antibody (1:5000 dilution; catalog no.: A8592; Sigma).

### Determination of cellular ROS

Dichlorodihydrofluorescein diacetate (Sigma‒Aldrich) can be used to mark the expression level of cellular ROS. Cultures of human GCs were spiked with 10 μM dichlorodihydrofluorescein diacetate for 30 min, followed by three washes of human GCs with PBS before capturing the fluorescence signal using a fluorescence microscope (Leica).

### Immunohistochemical staining

Ovarian tissues were serially sectioned using formalin fixation and paraffin embedding. The sections were dewaxed in xylene, treated with gradient alcohol, and subsequently treated with 3% H_2_O_2_ for 20 min. The antigen was repaired at high temperature with sodium citrate buffer (pH 6.0) and subsequently incubated in the blocking solution for 30 min. Primary antibodies were incubated overnight at 4 °C using primary antibodies as follows: cleaved caspase-3 (1:200 dilution; Cell Signaling Technology) or KLF12 (1:500 dilution; Santa Cruz Biotechnology). The sections were then washed three times with Tris-buffered saline, and secondary antibody was added. The sections were stained with 3,3′-diaminobenzidine and counterstained with hematoxylin. Control sections were incubated with a nonspecific rabbit immunoglobulin G antibody (53).

### Establishment of an ovarian oxidative stress mouse model

As previously described, an ovarian oxidative stress mouse model was established (54). Female ICR mice at 4 weeks of age were injected with 50 mg/kg 3-NP (Sigma‒Aldrich) twice daily at 12 h intervals for 5 days. About 12 h after the last injection, paired ovaries were removed and rinsed with PBS. The left ovary was snap frozen and stored at −80 °C for subsequent analysis of gene expression. The right ovary was fixed using formalin.

### *In vitro* culture of mouse ovaries

Both ovaries of 4-week-old female ICR mice were removed and washed three times in Waymouth medium containing 3 mg/ml bovine serum albumin. Ovaries (paired ovaries/well) were cultured on Millicell inserts (Millipore) in 24-well plates (Corning) with 600 μl of Waymouth’s MB752/1 medium (Gibco) supplemented with 100 IU/ml penicillin, 100 μg/ml streptomycin, 3 mg/ml bovine serum albumin, 10% fetal bovine serum, 1 mM sodium pyruvate, and 0.6 IU/ml rFSH (Merck). After 24 h of culture, the ovaries were infected with Ad-LacZ, Ad-FLAG-KLF12, and Ad-SPHK1 adenovirus (2.5 × 10^10^ plaque-forming unit/ovary) for another 48 h before the medium was changed. The ovaries were then cultured with 2 μg/ml 3-NP for an additional 48 h at 37 °C in a humidified atmosphere of 5% CO_2_. The cultured ovaries were collected for measurements of mRNA and protein expression.

### EMSA

To prepare protein extracts from human granular cells, homogenize granular cells in ice-cold cytoplasmic lysis buffer. The oligonucleotides of the SPHK1 promoter (5′-GAAGTCATAGTCATTGGCAGTGGGGCACAGGCTCTGCAG-3′) were synthesized and labeled with IRDye 700 (IDT). EMSAs were performed with Odyssey IRDye 700 infrared dye–labeled oligonucleotides using an EMSA buffer kit (Thermo Fisher). Incubate for 30 min at 30 °C. The gel was run for 90 min at 90 V. The specificity of the binding was examined using competition experiments and the gel supershift assay by adding KLF12 antibody (Abcam) prior to the addition of the fluorescently labeled probe.

### Statistical analysis

Data are expressed as the mean ± standard deviation. GraphPad Prism software (version 8.0; GraphPad Software, Inc) was used for statistical analysis. One-way ANOVA was used for experiments with more than two groups. *p* < 0.05 was statistically significant. At least three experiments were performed.

## Data availability

The original data presented in the study may be found in the article/supporting information section. Further inquiries can be directed to the corresponding author.

## Supporting information

This article contains supporting information.

## Informed consent

Informed consent was obtained from all subjects involved in the study.

## Conflict of interest

The authors declare that they have no conflicts of interest with the contents of this article.

## Supporting information

Supporting Table S1 The list of primers is shown in Table S1.
